# Current influences and approaches to promote future physical activity in 11–13 year olds: a focus group study

**DOI:** 10.1186/s12889-015-2601-9

**Published:** 2015-12-21

**Authors:** Angela Carlin, Marie H. Murphy, Alison M. Gallagher

**Affiliations:** Northern Ireland Centre for Food and Health, Biomedical Sciences Research Institute, University of Ulster, Coleraine campus, Cromore Road, Coleraine, BT52 1SA UK; Centre for Physical Activity and Health, Sports and Exercise Sciences Research Institute, University of Ulster, Jordanstown campus, Shore Road, Newtownabbey, BT37 0QB UK

**Keywords:** Physical activity, Focus groups, Adolescence, Barriers, Facilitators of physical activity

## Abstract

**Background:**

Many children and adolescents are failing to meet current physical activity (PA) guidelines and consequently not achieving the benefits associated with regular participation in PA, with girls consistently less active than boys. In order to design interventions to increase physical activity in adolescents it is important to understand their perceptions of and preferences for physical activity.

**Methods:**

One hundred eighty participants, mean (SD) age 12.1 (0.5) years, completed the Physical Activity Questionnaire for Children (PAQ-C) and had height and weight measured. This information was used to select a subsample of participants (n64; mean (SD) age 12.3 (0.4) years; 39 females; 25 males; 25 % overweight/obese) to take part in focus group discussions. Participants were grouped based on PAQ-C responses into ‘low-active’ and ‘highly-active’ groups, so that those with similar existing levels of PA were in the same focus group. A semi-structured discussion guide was employed to explore the key influences on current PA participation and to actively seek ideas on how best to promote future PA in this population. In total, nine focus groups (mixed-gender) were conducted within the school setting. All focus groups were audio recorded, transcribed verbatim and analysed thematically.

**Results:**

A number of themes emerged in relation to influences on current PA including friendship and peers, family and other people, the consequences of not taking part in PA, changing priorities, and cost and access to resources. With regards to the future provision of PA, participants favoured opportunities to try new activities, increased provision of school-based activities which can be undertaken with friends and activities which incorporated the use of technology and encouragement through rewards and incentives. Gender differences were apparent in relation to the types of activities participants preferred taking part in. Differences were also observed between ‘low-active’ and ‘highly-active’ groups in relation to barriers to current participation in PA.

**Conclusions:**

This study has highlighted a number of influences on current and future participation in PA, which differed based on gender and existing PA levels, for example, maximising the potential of the school day and including technology and incentives. These components can inform targeted interventions to increase PA in low active adolescents.

## Background

Children and adolescents are currently recommended to undertake at least 60 min per day of moderate to vigorous physical activity (PA) [1]. The physical and psychological benefits of PA in children and adolescents are plentiful [1, 2], however many young people are failing to meet current guidelines. On a global level, approximately one fifth of 13–15 year olds are meeting the current guidelines [3]. Similar trends were observed in Northern Ireland with only a quarter of children aged 9–11 years meeting the recommended 60 min per day of MVPA [4].

There is a need for policy makers to regularly review influences on PA in children and young people and to understand what helps and hinders them in relation to PA participation [5]. The transition from primary to post-primary education represents a key period of change [6] and typically represents the onset of declining PA behaviours [7, 8]. Exploring the issues related to current influences on PA, and comparing these differences between young people with varying existing levels of PA participation is therefore pertinent in this age group to further develop understanding of changing PA behaviours.

A review of qualitative studies highlighted the importance of social support from family and significant others in maintaining participation during key changes in the lifecycle, for example, school transitions [9]. The majority of included studies in young females cited barriers including negative experiences during physical education, dislike of uniforms or the sports offered, and the conflicting notion that being sporty did not make girls appear feminine and/or desirable to boys [9]. Boys were found to hinder girls’ participation through name-calling and negative opinions of girls who were active, describing them as ‘disgusting’ and ‘nasty’ [10]. More recent work confirms these findings as well as highlighting further barriers to participation in girls, for example, lack of time, loss of interest, practical issues, influence of peers and body centred issues [11, 12]. Friends have been frequently cited as influencing PA during adolescence [11–13], with young people belonging to a number of friendship groups that can influence both the initiation and maintenance of PA [14].

Given the evidence that activity indicators such as sport participation in adolescence may contribute to future involvement in PA [15], it is also necessary to examine how best to promote PA in this population, which is important to inform future PA interventions in younger people, particularly amongst the least active. While much qualitative work to date has examined the factors influencing participation and involvement in PA, less is understood about how to actively involve children and adolescents in planning PA interventions, what they prefer in such interventions and how to best promote future PA in this age group. Identifying barriers and facilitators to PA is key to understand the complex relationship between young people and PA [10] but it is also important to identify components to be utilised in future interventions aiming to increase PA in this group. Furthermore, owing to the greater use of focus groups which to date have been largely homogeneous with respect to gender, most commonly featuring females, there is a dearth of evidence on influences on PA derived from qualitative work that is mixed-gender. Given that at this stage of the lifecycle the school environment represents a key opportunity for modulating PA, and that many children attend co-educational schools, exploring the factors associated with participation in a mixed cohort may provide fresh ideas. Understanding the interactions between males and females and how this can influence PA participation will provide useful insight to inform the development of future interventions which may be targeted at mixed or single gender groups, albeit in a mixed-gender environment, for example, recess-based interventions taking place in the school playground. Although a number of influences on PA may be gender specific at this stage of the lifecycle, children at this age are unlikely to feel as averse about sharing their views and opinions in front of other peers [16] compared with those at a later stage of adolescence.

The aims of this study were to investigate key influences on current levels of PA and to explore possible ways of increasing participation, for example, identifying components which can be utilised in future interventions in children and adolescents by actively seeking ideas from this target population [17, 18].

## Methods

Focus groups are widely employed in qualitative research and can be used within many areas of health research to provide detailed information on the range of feelings and ideas individuals experience in relation to a particular issue or behaviour [19]. The open-ended approach provides a useful method for exploring, in depth, the factors associated with PA in this age group [20]. This study was approved by the University of Ulster Research Ethics Committee. Written consent was obtained from parents/guardians and children.

### Sample selection

A convenience sample of schools in Northern Ireland (*n* = 5) were invited to take part in the study via e-mail/ telephone. Following permission from school principals, invitational letters, including information about how to take part in the study, were sent to parents/ guardians of all pupils (*n* = 300) aged 11–13 years attending 3 post-primary schools in Northern Ireland (2 schools declined to participate). Each post-primary school was located in a different district council area within Northern Ireland. All participating schools were co-educational (2 Grammar schools and 1 secondary school). Socioeconomic status of individual participants was not measured however the selected schools had a wide catchment area and all three reported their student body reflected a range of socioeconomic statuses and participants from both urban and rural environments. Those pupils who returned consent forms from parents/guardians and assent forms were eligible to take part.

### Procedure

#### Phase 1

One hundred eighty participants (56 % female) completed the Physical Activity Questionnaire for Children (PAQ-C) [21] and a Health and Lifestyle questionnaire [22]. The PAQ-C questionnaire [21] captures information on PA participation over the last 7 days in children while the Health and Lifestyle questionnaire [22] captures information on PA participation and other health related factors, for example, where children live, how they travel to school and how they view their PA/health compared to their peers.

Participants were asked to identify any existing medical conditions that they felt were related to their involvement in PA and that they may not feel comfortable talking about in focus group discussions. These participants were subsequently excluded from participating in phase 2 of the study (*n* = 18). Each participant had height and weight measured to the nearest 0.1 cm and 0.1 kg respectively using a free standing stadiometer (Leicester Height Measure, Marsden Group) and digital scales (Seca 877). These were used to calculate body mass index (BMI) (kg/m2). PAQ-C questionnaires were scored to provide a composite score for PA ranging from 1–5 (where 5 is the most active), which was calculated by taking the mean score of the 9 items used within the questionnaire [21].

#### Phase 2

Participants were ranked from least active to most active based on PAQ-C scores (males and females were ranked together) and a subsample of participants representing the top and bottom tertiles of PA scores (*n* = 64; 39 females; 25 males) were invited to take part in focus group discussions. Participants were allocated to a focus group with other participants based on having a similar PAQ-C score. Participants were allocated to a focus group with other participants from their own school. Each focus group had five to eight participants, with three groups of ‘highly-active’ participants (mean PAQ-C score 4.2/5) and six groups of ‘low-active’ participants (mean PAQ-C score 2.6/5). A larger number of ‘low-active’ groups were selected to increase the volume of data generated from the least active participants, who are likely to be a target population in future PA interventions. Composing focus groups on the basis of current PA levels was beneficial for a number of reasons, namely being able to categorise groups as ‘low-active’ or ‘highly-active’ enables responses between such groups to be compared in relation to PA levels. Furthermore, by grouping participants with peers of similar PA levels, it was likely that these participants had similar experiences in relation to activity and would therefore perhaps feel more comfortable talking about them. In addition to PAQ-C scores, participants’ BMI was also taken into consideration to ensure a representative sample from each weight category was included within each focus group, i.e. that the proportion of participants within each weight category in each focus group discussion was reflective of the population average for this age group. This ensured that the views of participants who were underweight, normal weight or overweight/obese were all represented within each focus group discussion.

All focus group discussions were facilitated by the same moderator, and an assistant moderator was also present to make notes to aid subsequent data analysis. Saturation was achieved during focus group nine and no further participants were selected to take part in focus group discussions.

### Focus group topics

All focus group discussions followed a semi-structured discussion guide (Table 1), with topics derived from reviewing the existing literature in relation to PA in this population. The discussion guide was not informed by theory. The semi-structured nature of the topic guide ensured the same key areas were explored within each discussion, whilst still allowing flexibility within individual group discussions. During the opening question of each focus group discussion, the moderator highlighted to participants that PA may refer to “sports or dance that make you sweat or make your legs feel tired, or games that make you breathe hard, like tag, skipping, running, climbing, and others”, consistent with the definition given to participants when completing the PAQ-C questionnaire [21]. Open-ended questions were employed to stimulate conversation and probing questions were used to further explore the comments made by participants, for example, “In what way do you mean?” or “Could you explain that further?”

**Table 1:** Semi-structured discussion guide for focus groups

Focus groups	
What are your favourite types of physical activity?	
What do you think are the benefits of being physically active?	
How do you feel when you take part in physical activity?	
What factors influence your own physical activity? What people?	
What are the barriers towards your participation in physical activity?	
What factors or approaches would encourage you to participate, or participate more, in physical activity?	
How do you feel about walking as a form of physical activity?	
How do you feel about the role of technology/mobile phones/ applications in physical activity?	
Are there any other thoughts or ideas you would like to share that haven’t previously been covered in today’s discussion?	

### Focus groups

All focus groups were conducted in the school setting as it was an environment in which participants were comfortable with. Focus group discussions took place during school hours and each focus group was conducted in a classroom, with chairs arranged in a circle. Ice-breakers and introductions were used in an effort to make participants more familiar with the discussion and to put them at ease. Following explanation of the procedure, verbal consent was obtained from all participants, in addition to the written consent obtained at recruitment, to ensure they still felt comfortable participating. Within discussions, efforts were made to ensure all children participated, for example, making eye contact with participants to encourage them to contribute to the discussion. To maintain the interest and enthusiasm of participants, each focus group took approximately 50 min to complete, with the actual discussion not lasting more than 45 min [16]. All participants were provided with refreshments following completion of the focus group discussions.

### Data analysis

All focus group discussions were audio-recorded and transcribed verbatim. Data were analysed thematically, using a deductive approach which involved the following six key phases [23]. Familiarisation with the data was achieved by listening to the audio-recordings and re-reading transcripts. Each transcript was then subjected to systematic coding conducted by a member of the research team (AC), whereby meaningful quotes or key examples from participants were assigned a code. Potentially relevant codes were then grouped together to develop themes. These themes were then reviewed by a member of the research team (AC) to ensure the themes were representative of the coded excerpts. Once themes had been reviewed throughout the entire data set, definitions and names were then formally assigned to each theme. The process of coding and reviewing themes was repeated independently by a second member of the research team (AMG) to minimise the potential for bias and to ensure that all quotes were correctly coded. It was agreed that data saturation had been achieved when no new codes materialised from the final two transcripts. Quotations from participants were used to highlight typical responses and ideas that led to the development of key themes. In order to differentiate between the different characteristics of participants, quotes will be followed by a short key, for example, (G, LA) indicates a female participant from a ‘low-active’ focus group, while (B, HA) demonstrates the quote was from a male participant in a ‘highly-active’ group. IBM SPSS (version 20) was used to analyse anthropometric data and quantitative data from questionnaires which enabled categorisation of participants as ‘low-active’ or ‘highly-active’ and subsequent selection of the focus group sample.

## Results

### Participant characteristics

Of the 64 selected for invitation, 62 (97 %) participants took part in focus group discussions; 2 participants were absent on the day of the focus group. The characteristics of this sub-sample of participants are presented in Table 2. Mean (SD) age of participants was 12.1 (0.50) years. 26 % of participants were overweight or obese [24]. Of the 62 participants who took part in this stage of the study, 84 % self-reported that they enjoyed PA with 92 % stating that they enjoyed physical education and games class. Approximately half of participants (52 %) felt their time spent in PA during leisure time was ‘the same’ compared with others of their age. Participant responses below highlight the key themes and are categorised under two main headings; influences on current levels of PA and how to increase participation in PA.

**Table 2:** Characteristics of focus group participants

Characteristic	All participants (*N* = 62) n (%)	Low-active participants (*N* = 39) n (%)	Highly-active participants (*N* = 23) n (%)
Gender			
Female	24 (38.7)	14 (35.9)	10 (43.5)
Male	38 (61.3)	25 (64.1)	13 (56.5)
Mode of travel to school			
Bus/car/train	52 (83.9)	35 (89.7)	17 (73.9)
On foot	10 (16.1)	4 (10.3)	6 (26.1)
Live			
Town or city	25 (40.3)	13 (33.3)	12 (52.2)
Village or countryside	37 (59.7)	26 (66.6)	11 (47.8)
BMI category^a^			
Underweight	4 (6.5)	2 (5.1)	2 (8.7)
Normal weight	42 (67.7)	23 (59)	19 (82.6)
Overweight	12 (19.4)	10 (25.6)	2 (8.7)
Obese	4 (6.5)	4 (10.3)	0
Ever tried to lost weight/avoid weight gain?			
Yes	39 (62.9)	25 (64.1)	14 (60.9)
No	23 (37.1)	14 (35.9)	9 (39.1)
If yes, how?			
Diet from doctor/dietitian	2	1	1
A diet made up/found themselves	14	10	4
Doing more exercise	33	19	14

^a^International Obesity Task Force cut off points [24]

### Influences on current levels of PA

1. Friends and peers
  The influence of friends and peers was the most commonly recurring theme, irrespective of PA levels (Table 3). Many participants linked their own participation in PA to what their friends were doing, highlighting they would only take part in PA if they knew people or their friends were also going to take part. The opportunity to make new friends was also an influence on current activity: *“You meet new people. So you have like different friends in the different clubs that you go too”* (G, HA). In contrast, peers could also have a negative influence on PA participation, with participants feeling conscious about how others viewed them when they were being physically active (Table 3). This was most evident amongst low-active female participants: *“If you’ve done it, this activity, this sport and someone else has said, has like laughed at you doing it or like said you’re really bad, then you wouldn’t want to do it again, you’d just embarrass yourself”* (G, LA). Girls were also more likely to cite how getting older had changed the attitudes of people around them to PA, which was seen as an influence on their participation*: “If you were a girl, it wouldn’t be the thing to go up and like play in the courts or anything like we used to do. We couldn’t do that cause like loads of other people would think, like it’s kinda being frowned upon*” (G, LA). Such views were not shared by male participants. Low-active participants were more likely to negatively view other classmates who were more active than them, citing that such people often flaunted the fact they were more active and made people feel uncomfortable with their competitiveness (Table 3).

**Table 3:** Factors that influence current levels of physical activity

Theme	Quote
(1) Friends and peers	“You’d probably make more friends when joining your clubs and things” (G, LA)
	“Erm like my friends go to football and all that as well, and I would hang out with them and they play football all the time as well- so it helps me” (B, LA)
	“Aye like I wouldn’t do a lot of stuff, if I was playing like a football match or whatever, I wouldn’t want to play against like another team cause I’d be really worried, like I’d be really worried about what they think of me” (G, LA)
	“She can be like really like competitive; she can like hurt other people by being that competitive. Like saying aw I’m gonna win, and then she would flaunt it in peoples’ faces and stuff” (G, LA)
	“There are some people who are too physically active and like to sort of shove it in your face” (B, LA)
(2) Family	“My like sister and my like mum are always like pushing, my mum like pushes me to do like different sports” (G, LA)
	“Be good sometimes if they did that sport sometimes they’d be like forcing you to do it and sometimes you’re good at it so they’re encouraging you to do it” (B, HA)
	“My mum would go out walking and she’s always encouraging me to go out walking as well” (G, LA)
	“Well if you have a family member or someone who is very unactive, they don’t hardly do anything, it might encourage you that it would be a great idea to do something because they’re not doing anything, and you notice how, I suppose, how unhappy or something they are and how it’s affecting them not doing anything so then you would want to do it so you don’t end up like” (B, LA)
(2) Other people	“See I’d be the type being really, you know like saying ‘aw I can’t do it, what’s the point in doing it if I can’t do it’. So then she’d be like, tell me be really positive, tell me ‘aw you can, you may as well just try’” (G, LA)
	“Well when you see them on TV like sometimes you might think that you want to be as good as them someday” (B, HA)
	“Like if they’re good at it, like it makes it look good then so you want to be as good as them” (B, HA)
	“Friendly teachers, friendly and good teachers that will teach you how to do it well I suppose, and they don’t force you to do it, and I suppose more welcoming if you haven’t done it before and your bad at it” (B, HA)
(3) Consequences of not taking part	“It means you generally eat more, because you’re sitting in the house and you want to do something so you go eat something” (G, LA)
	“You’d be very unsociable, say like you’d just kind of want to stay in the house all the time and not really do anything, like go outside or anything” (G, HA)
(4) Changing priorities	“I used to like go to a lot of after school things, I don’t really like after school things as much anymore because I’m really tired at the end of the day and I don’t want to go and do more physical activity” (G, LA)
	“Or there’s other things that you would want to do and prioritise and then, you know, activity just falls off the list” (B, LA)
(5) Cost and access to resources	“I don’t, I don’t know whether this is right or not but I think people would probably be more attracted to it if it was quite cheaper or it was free” (G, LA)
	“Erm like stuff… that are kinda near to you like, really the only real thing near to me is football but I don’t really like it so” (B, LA)
	“Cause then you can try it and if you don’t like if you don’t like it but you have to buy stuff for it then you feel like it’s a waste of money” (G, LA)

G: Girl; B: Boy; LA: Low-active; HA: Highly-active
2. Family and other people
  Family remained an important influence on PA, with participants commenting that their parents/ guardians often provided them with verbal encouragement to be more active (Table 3). In addition, a number of participants explained that family could indirectly influence their PA behaviours, by observing what their older relatives were doing and not wanting to end up like them. Low-active female participants highlighted that their parents often encouraged them to participate in activities with them, for example, walking (Table 3). While parents were frequently cited as positively influencing PA, both by encouragement and practically (providing transport to activities and providing equipment), some young people stated they no longer felt comfortable taking part in activities such as playing in the park with their parents. This was more common amongst highly-active participants, with many stating they would feel childish and not want their friends to see them: *“You kind of feel like you’re a bit more baby-ish to be hanging about with your mum and that instead of going on your own to the park or something”* (G, HA).
  The influence of role models was more commonly quoted by highly-active participants, with many stating that observing what famous sports stars or Olympians were doing made them more determined to succeed at their chosen sports. Coaches and managers were also influential, with participants explaining the importance of an approachable, friendly coach who didn’t shout at them in keeping them interested and having fun at their chosen sport: “*If you had a nice coach or something - they don’t act like a coach, they act more like a friend to you instead* (G, HA).*”*
3. Consequences of not taking part
  This age group were particularly aware of the consequences of not leading a physically active lifestyle and many highlighted this as a factor which encouraged them to be active. Across all groups, participants cited a number of health conditions they would be worried about if they didn’t do enough activity: *“Like I always think things, like if I didn’t do like sports I would get really sick and have like heart problems and like become like obese and stuff like that”* (G, LA) and the impact being inactive could have on their weight *“You’d get all fat”* (B, LA). Emotional consequences were also discussed, including the negative effects being inactive could have on mood: *“It takes your mind of something, you know it helps you relax as well”* (G, LA), while not taking part was also highlighted as a way of isolating yourself from friends and becoming unsociable (Table 3). In addition, by not spending time in PA participants reported that this may increase time spent in other unhealthy behaviours, with a number of low-active participants expressing that they would be more likely to eat when they are bored and not doing activity (Table 3).
4. Changing priorities
  Participants frequently cited changing priorities as a barrier which influenced their participation in PA. Many low-active participants felt they spent less time in PA as they had gotten older: “*I think I did more in primary school, ‘cause like our teacher was very active and she like took us outside and stuff”* (B, LA). Increased barriers cited by female participants reflected the recent transition from primary to post-primary education and included increased time studying and longer commutes to school limiting free time available for PA. Both male and female participants highlighted how wanting to spend free time doing other things, for example, watching TV limited the time they had available for PA, with these barriers were more frequently cited by low-active participants (Table 3), with highly-active participants more likely to share the opinion that moving to a bigger school with more facilities had provided them with more opportunity to be active: *“I would say more cause there’s like more things to do and because you want to try new* sports” (B, HA).
5. Cost and access to resources
  The availability of activities, equipment needed to participate and cost were all frequently highlighted as influences on current participation. Not surprisingly, sports and activities that were free and required few or no resources to participate were more appealing to this age group. In addition, access to activities outside of school was problematic as many young people had to rely on parents/ guardians for transport: *“If there wasn’t really, like if you didn’t know, if there wasn’t any clubs around you that you wanted to take part in and you were too far away, like petrol and all that, the time to get there and things like that”* (G, HA). Weather was frequently cited as an influence with many participants highlighting that good weather encouraged them to be more active: *“If it’s sunny and warm it’s nice to get out”* (G, LA). Low-active female participants were more likely to cite weather as a barrier to PA: “*I suppose like the weather, like if it’s raining I wouldn’t go for like a run or whatever”* (G, LA), however this was not consistent in low-active males or in highly-active participants: *“I like going out on my bike when it’s wet and I like playing, I like playing rugby matches when it’s wet cause it’s like more enjoyable, the grounds nice and dirty”* (B, HA).

### How to increase participation in PA

1. Try new activities
  When asked how best to promote activity in this target group, the provision of new activities was commonly cited within the focus groups. Irrespective of gender, most low-active participants highlighted the need for new activities to be provided*: “Or like something like different to like normal, we do tennis and running a lot, something a bit different. You’d just get sick of the one sport”* (G, LA). The majority of low-active female participants cited they wanted a move away from activities that were usually offered, which were structured or team-based in nature: *“Something different; like not Camogie*^1^*or football because you can do that anywhere, just join like a team, but like something you can’t do like with your team or whatever, like something interesting”* (G, LA). Instead, this sub-group felt activities that were more informal and fun would be more promising: “*Maybe if they weren’t all sports because some people don’t like always like do a particular sport because you have to actually be good at it. But maybe if there was something to do with like not sporty but it still encouraged you to get outside and do something”* (G, LA). Examples of informal activities included social events such as sponsored walks, litter picking walks on local beaches, dance classes and water sports.
2. Increase school based activities with friends
  This age group felt increasing the provision of activities within school would be an effective means of getting more young people involved in different activities. A number of reasons were cited in favour of schools being a good environment to promote PA, for example, at break and lunchtimes: *“It would give you a chance to relax if you did it in the middle of the day, relax before the final classes and it would give you a chance I suppose to talk to people and catch up and stuff like that, I dunno*” (B, LA). Furthermore, the school setting was viewed as beneficial since participants had an existing peer network that they could take part in activity with: *“In school because you could like do stuff with your friends, like “aw are you going to go to it” and then loadsa people would go, it would just be more fun”* (G, LA). Having friends take part was an important means of increasing participation amongst low-active participants: *“Like if my friends weren’t going I wouldn’t go”* (G, LA). Not having friends present may also discourage young people from returning: *“Cause if you’re sitting there by yourself and you don’t know anyone it’s a bit awkward and you don’t want to go again cause you didn’t have any fun”* (G, LA). Most participants were happy to attend activities that were mixed-gender, with some highlighting it gave girls the chance to do other sports: *“If you’re doing football and there’s only one girl that wanted to do it and the rest was boys, and you weren’t allowed cause it’s just boys, so maybe if it was like a mixed one”* (G, HA). In addition, girls in particular highlighted the negative aspects if the sport was single gender: “*If it kinda was the same all girls all the time, you’d just get into cat fights”* (G, HA) and the added enjoyment if activities were mixed gender: *“Some girls would want to be with boys in the other group…the boys would have to do it. Boys are more craic*^2^*” (G, LA).* However boys were often quick to highlight mixed-gender might not be appropriate for all activities: *“When you’re playing rugby you couldn’t have the girls there*” (B, HA) and were more likely to view certain sports as gender specific: *“Probably different, the boys at one thing and girls at a different, cause girls don’t like football”* (B, HA).
3. Include technology
  Many participants felt that technology could be incorporated into PA initiatives to engage young people and make it more interesting: *“It would interest people if there was technology involved really, it wouldn’t just be normal”* (B, HA). Both low-active and highly-active participants felt technology had a role to play in making young people aware of what they should be doing*: “‘cause you could have how many someone of a certain age should be doing and then you get to see how many you are doing”* (B, LA). In addition, many low-active participants felt technology could be useful when setting goals to be more physically active: *“It would try and make you work harder, to try and get the goal. ‘Cause like when you can see it in black and white you’ll know then what you have to achieve”* (G, LA). While all participants felt technology was useful for self-monitoring, those in the highly-active group were more likely to relate this monitoring to the idea of competing against others: *“It would make me want to do more to try and beat your own record or other people’s records”* (B, HA).
4. Provide rewards and incentives
  Many participants felt it was important to provide rewards and incentives to engage young people in new activities: *“More people would want to take part because if they knew that they could get a reward, they’d just want the reward so they would take part”* (G, LA). In addition, providing incentives could encourage adherence to PA and help people work towards goals: “*Yeah like if you walk a certain amount of steps you get a certain amount of points, and then the number of points or something adds and you can get whatever you want, like this certain thing costs 40 points or something”* (G, LA). Low-active participants in particular reported that it was important that such rewards were provided on the merit of effort and not just to those who excelled at particular sports: *“You got points for putting the effort into it, like even if you’re not the best person that you still tried your best.* (G, LA)*”.* Examples of suitable rewards differed by gender with females highlighting vouchers for shops and cinema trips as suitable rewards whereas male participants preferred sporting equipment and trips related to activity, for example, going to watch a football game.

## Discussion

This study aimed to explore the main influences on current PA participation and how best to engage adolescents in PA immediately following the transition from primary to secondary school (11 – 13 year olds). The focus groups identified several issues which will be important to consider in the design of future interventions to be targeted at this age group.

Friendship and the influences of peers were consistent themes both for influences on current activity and adolescents’ suggestions on how to promote participation. This theme emerged irrespective of current PA levels and underlines the importance of peers at this stage of the lifecycle [25]. The influence of friends and the importance of having someone to participate with have been shown to be key factors in young females’ participation in PA [11, 20, 26, 27]. The present study also suggests that this is equally important amongst males. Gender differences were observed, with girls, especially those who were less active, more likely to cite the negative influence of peers on their levels of PA and how this made them feel conscious about participating in PA in front of others. These findings are supported by previous qualitative work, where young females reported feeling their sporting ability was judged by other girls [12] and highlights the potential influence of peer victimisation on adolescent PA [25].

Participants cited the important role parents play in their involvement in PA. Parental support and direct help from parents have previously been correlated with PA in adolescents [28, 29]. Given the continued influence parents play as children move into adolescence [29], it is important to incorporate some level of family support into future interventions. Furthermore, the role of parents may be better suited to supporting young people in activities that they can participate in with friends given that highly-active participants were conscious that participating in activity with parents may be viewed as ‘babyish’ by their peers. Parents have been previously shown to negatively influence PA in this population, by actively discouraging participation [12], not wanting their children to get injured from sport [10] or placing decreased emphasis on sports participation over other commitments, for example, studying [11]. These negative influences were not apparent within the present study.

Both male and female participants had a strong awareness of the health benefits of PA however this awareness did not necessarily translate into PA related behaviours amongst those who reported the lowest PA levels based on their PAQ-C responses. Most of the literature to date suggests that most facilitators of children’s PA participation are focused on the ‘here and now’ [17]; with little focus given to the impact of PA participation on adult health as a motivating factor [17]. Promoting awareness of the health benefits of PA may be an effective means of engaging young people in PA [18], however previous work has not evidenced such an awareness in participants aged 12 to 14 years [18]. This data suggests that awareness of the benefits of an active lifestyle may be increased in this particular population compared with others of a similar age however it is unclear where this increased awareness has come from, for example, schools, media or family. While young people may already be aware of the benefits, it may be important that future interventions reinforce these as they may act a potential motivator [30].

Moreover, evidence has highlighted that reinforcing specific health benefits of PA, for example, the positive effect it may have on body image, can increase motivation for being physically active, particularly amongst adolescent females [30]. Over half of participants in the present study reported trying to lose weight in the past through exercising, which was reflected further in focus group discussions, and is consistent with previous studies in female only cohorts [20]. While direct links were not drawn between exercise and body image [20], it is likely that references to weight gain and obesity were more closely linked to body image as opposed to the consequences of overweight/obesity on health.

Within the present study, highly-active participants felt the transition to secondary education provided further opportunity to be active given the increased activities on offer, including lunchtime and after-school training and games. Contrastingly, the transition from primary to post-primary education and the associated decrease in time available for participating in PA was a key barrier for low-active participants. Lack of time has previously been identified as a barrier to exercising amongst adolescent females [31], with males more likely to state ‘wanting to do other things with my time’ as a barrier [31]. Within the present study, both genders indicated the desire to spend more time doing other things over PA.

Homework and other factors that may limit free-time for activity after school highlights the importance of maximising PA participation within the school day to overcome these barriers, particularly for low-active participants. Timetabled physical education classes may be one of the few opportunities adolescent girls have to be active [10] and this may also be apparent for low-active boys within the present study. Physical education has the potential to contribute towards time spent in MVPA [31, 32] however further consideration should be given to the preferences of young people in terms of the types of activities they want to participate in. The present study identified gender differences, with male participants preferring competitive, intense forms of PA, usually comprising structured sport, compared with females who preferred new activities such as dance. A preference for activities that were unstructured in nature amongst low-active females is consistent with previous findings that activity should be informal in nature [20].

A whole school, multicomponent approach to the promotion of physical activity is key [5] and was highlighted as one of seven key investments that work for PA [33]. Within this framework, one main priority was for the provision of a suitable environment and adequate resources to facilitate both structured and unstructured PA throughout the school day [33]. In addition to the provision of equipment, schools have the ability to reach all youths, irrespective of their socioeconomic status or background [34]. Participants in this study felt schools provided an ideal environment for helping them become more active, outside of physical education. The extracurricular activities currently offered within schools tended to reflect the content of timetabled physical education, which has been evidenced in previous studies [35]. Increasing further opportunities to be active could maximise the potential of the school day in helping young people meet the guidelines, especially those who are least active. Future initiatives could include the provision of recess-based activities and after-school programmes providing opportunities for all and not just a select group of skilled pupils [36].

Active playtime during the school day, i.e. during break and lunchtime, has the potential to contribute up to 40 % of daily recommended PA amongst children [37]. Despite this, there is currently a paucity of evidence on the effectiveness of recess-based interventions, particularly amongst adolescents [38] and is therefore an area that warrants further research. Given that females within the present study were more likely to highlight the social benefits that increased PA during the school day would have, in relation to having existing support from friends and peers, future initiatives should further facilitate these peer relationships. Identifying ways to increase social support for PA, particularly from peers, should be a priority for schools when trying to promote PA during school recess [39], for example, through peer mentoring schemes [20, 40, 41].

Walking was discussed an option to promote PA; to gauge interest in particular from low-active participants due to the limited evidence to date on interventions in this age group [42]. Walking presents a suitable activity that may overcome some of the frequently cited barriers to PA participation in this age group [9]. Many participants didn’t view walking as a form of activity; it was more something that was part of everyday life. This is reflective of previous work focused on walking behaviours, where it was viewed as opportunistic form of activity as opposed to intentional [43]. Sponsored walks and beach walks were highlighted by participants as informal ways to promote PA amongst adolescents. Findings from the present study highlighted that walking may present a useful means of engaging the least active in activity and provide them with a platform to build and subsequently become involved in other activities.

The school environment would also provide young people with the opportunity to participate in both single-gender and mixed-gender extra-curricular activities. Contrary to previous findings [44], the present work highlighted that girls, in particular highly-active girls, may enjoy taking part in mixed-gender activities. The mixed-gender nature of the focus groups may have contributed to this finding with female participants wanting to present themselves as ‘sporty’ to impress male members of the group. Clear gender perceptions existed from boys about what sports girls can and can’t do and appeared to be an ingrained attitude within male participants, which is consistent with previous findings from focus groups with boys [10]. Although females may enjoy participating in PA with their male counterparts, the different activities suggested by females that may increase their participation in PA, in particular the low-active females, highlights that a ‘one size fits all approach’ may not work for engaging this age group in further PA. Future initiatives may wish to provide opportunities for both mixed-gender and single-gender participation in PA.

The use of technology may also be important in future interventions with participants highlighting technology could make activities seem more appealing by increasing the novelty factor. ICT based technology in combination with other approaches can have a positive effect in promoting activity [45]. Given that 80 % of 15 year olds within the UK presently own a smartphone [46], developing mobile applications to work alongside PA interventions in terms of facilitating self-monitoring, goal setting and competitions amongst friends may be effective.

Rewards and incentives were also highlighted as a means of engaging young people in PA, and have been previously shown to increase levels of PA in youth [47, 48]. There is debate within the literature on how the use of rewards works to elicit behaviour change in individuals [49] however recent evidence has highlighted the effectiveness of financial incentives over usual care in adult populations [50]. Reward schemes have previously been shown to be highly acceptable for the promotion of healthy eating behaviours amongst adolescents [51]. This research has highlighted the importance of how rewards are implemented; it is important that all efforts are rewarded and not a competitive environment where only the sportiest achieve prizes. Furthermore, gender differences were highlighted with males preferring rewards that were linked to PA, for example, sporting equipment and trips to sporting events.

This focus group study exploring factors related to PA is the first to be conducted within a Northern Ireland population. Given the high proportion of youth inactivity in the UK, and that children living in Northern Ireland are those least likely to meet the current PA guidelines [52], this study provides great insight into how to best influence PA involvement in this population. When designing interventions, the involvement of target populations in the initial development of ideas is important [5]. As well as exploring current influences, this study explored how to increase participation and offset reported declines in PA in this population which may provide a focus for future PA interventions in this age group. A number of qualitative studies in this area have relied on school staff to select pupils, which may introduce bias, particularly in relation to how teachers view pupils’ activity levels. Within the present study, participants were selected for focus groups based on a validated measure of PA [53]. In addition, anthropometric data was collected to provide descriptive information on the population studied. 25 % of focus group participants were overweight/obese, which is representative of the Northern Ireland population [54]. The present study is one of the first studies to explore attitudes towards technology as a means of promoting or maintaining PA in this age group.

In contrast to previous work, participants were not assigned to focus groups based on gender. By having mixed-gender discussions, this study enabled the researchers to explore some key gender differences between boys and girls, and to facilitate interaction between participants with group members sharing similar or conflicting ideas on the key themes that emerged from group discussions. Although schoolchildren may be averse to sharing opinions on certain topics with participants from the opposite sex [55], it was felt that the target age group in the present study were not yet at the stage of adolescence where they would feel conscious about honestly and openly sharing their views and opinions in front of their peers [16]. Mixed-gender groups may have suppressed some gender-specific discussions about PA which have been identified by previous qualitative studies [10, 11] however it was useful to explore these areas in a mixed-gender environment as future PA interventions may be targeted at such environments, for example, the school playground. The use of focus groups which are homogeneous in respect to gender may be useful in future research targeted at a similar population to confirm if the key themes identified in this study are consistent to those generated from single gender discussions.

The limitations of self-reported PA are well documented [56], however using a validated subjective measure of PA to link with individuals’ responses provided weight to the themes, and is stronger than previous studies where children or teachers simply described participants as active without the use of validated tools or measures. The findings of the present study are specific to those who took part and may not be generalizable to other geographical areas however including participants from a range of PA levels increases the generalizability of the results.

## Conclusions

This study has highlighted a number of key differences between genders and existing PA levels in relation to current influences on physical activity and how best to promote activity. A number of gaps in the literature were explored, including the potential roles of walking and technology to promote PA. A range of possible intervention components were identified from focus group discussion that this age group believed would have a positive influence on young people engaging in future PA promotion initiatives. Utilising the school day, increasing the variety activities offered to adolescents, and incorporating technology and rewards within interventions may warrant further investigation. While previous work has highlighted the need for interventions to be tailored by gender, it may also be imperative that interventions are tailored to groups based on their existing PA levels. The findings of this study provide future direction for research in adolescents, as well as those working to promote PA within education, the community and at government level.

## Abbreviations

BMI: Body mass index
PA: Physical activity
PAQ-C: Physical activity questionnaire for children

## Footnotes

1 ‘Camogie’ is an Irish stick and ball team sport played by females

2 ‘Craic’ is an Irish word for fun/enjoyment
